# Supplementary figures and images for: Off-target sequence variations driven by the intrinsic properties of the Cas–sgRNA–DNA complex in genome editing
Source: PLoS One. 2025 Jul 18;20(7):e0328905. doi: 10.1371/journal.pone.0328905 (PMC12273960; doi:10.1371/journal.pone.0328905)

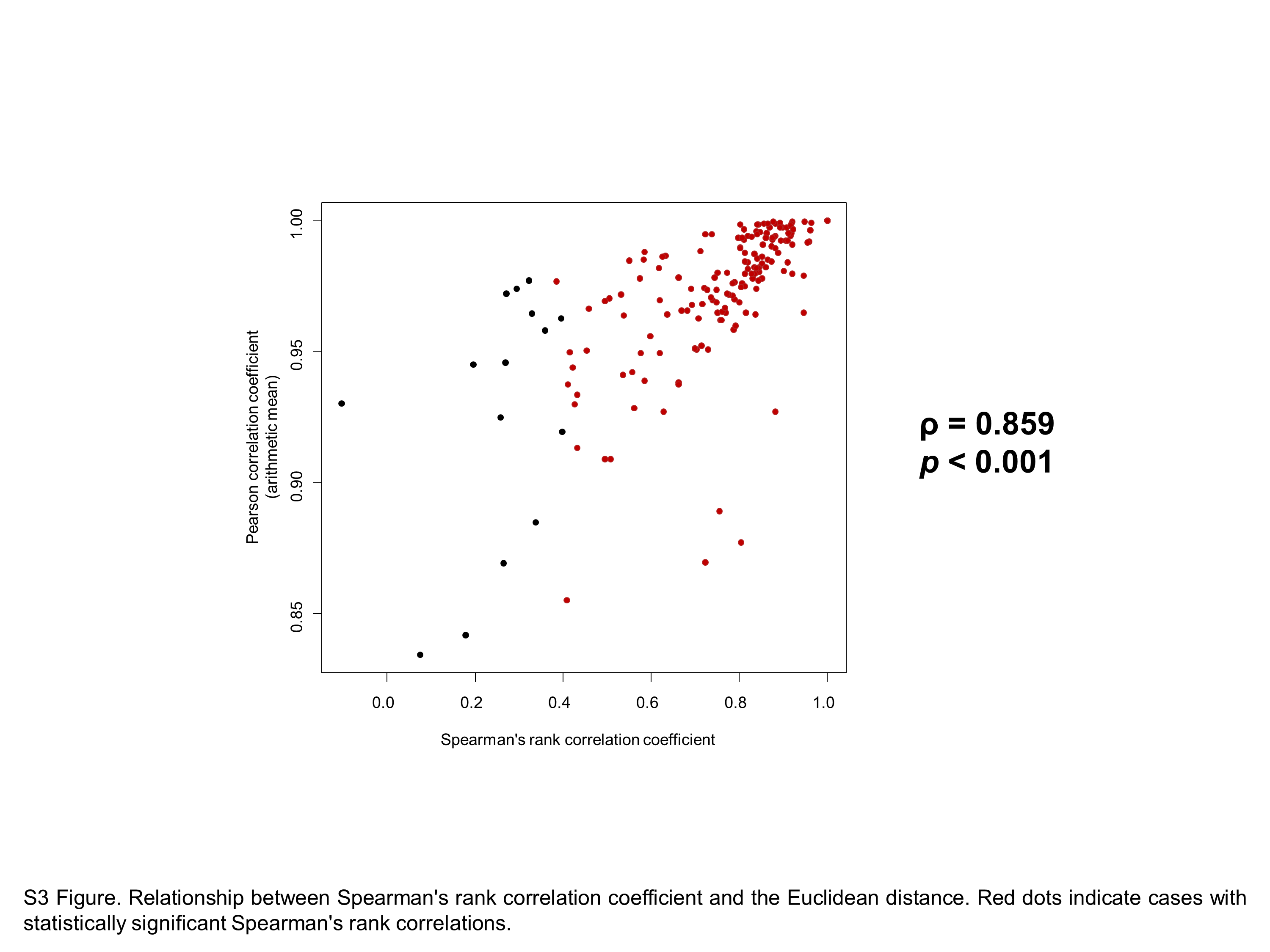

Supplement: S1 File — S1 Fig. Bar plots showing the number of off-target sites predicted exclusively by CRISPOR (FP, false positives), identified solely by GUIDE-seq (FN, false negatives), and shared between both methods (TP, true positives). S2 Fig. Distributions of CRISOT scores for the FP, TP, and FN groups. The mean and standard deviation for each group are shown. S3 Fig. Relationship between Spearman’s rank correlation coefficient and the Euclidean distance. Red dots indicate cases with statistically significant Spearman’s rank correlations. S4 Fig. Relationship between the Euclidean distance and the Pearson correlation coefficient. Red dots indicate cases with statistically significant Spearman’s rank correlations. S5 Fig. Spearman’s rank correlation coefficients for sequence patterns surrounding the VEGFA2 and VEGFA3 target sites across various Cas enzymes. All correlations were statistically significant with small EDs (<0.2). S6 Fig. Spearman’s rank correlation coefficients for sequence patterns surrounding the EMX1 and VEGFA3 target sites using WT SpCas9 across three different studies and cell lines. All correlations were statistically significant with small EDs (<0.2). (ZIP) [file pone.0328905.s001.zip › suppl_figures/F3_Fig.TIF]

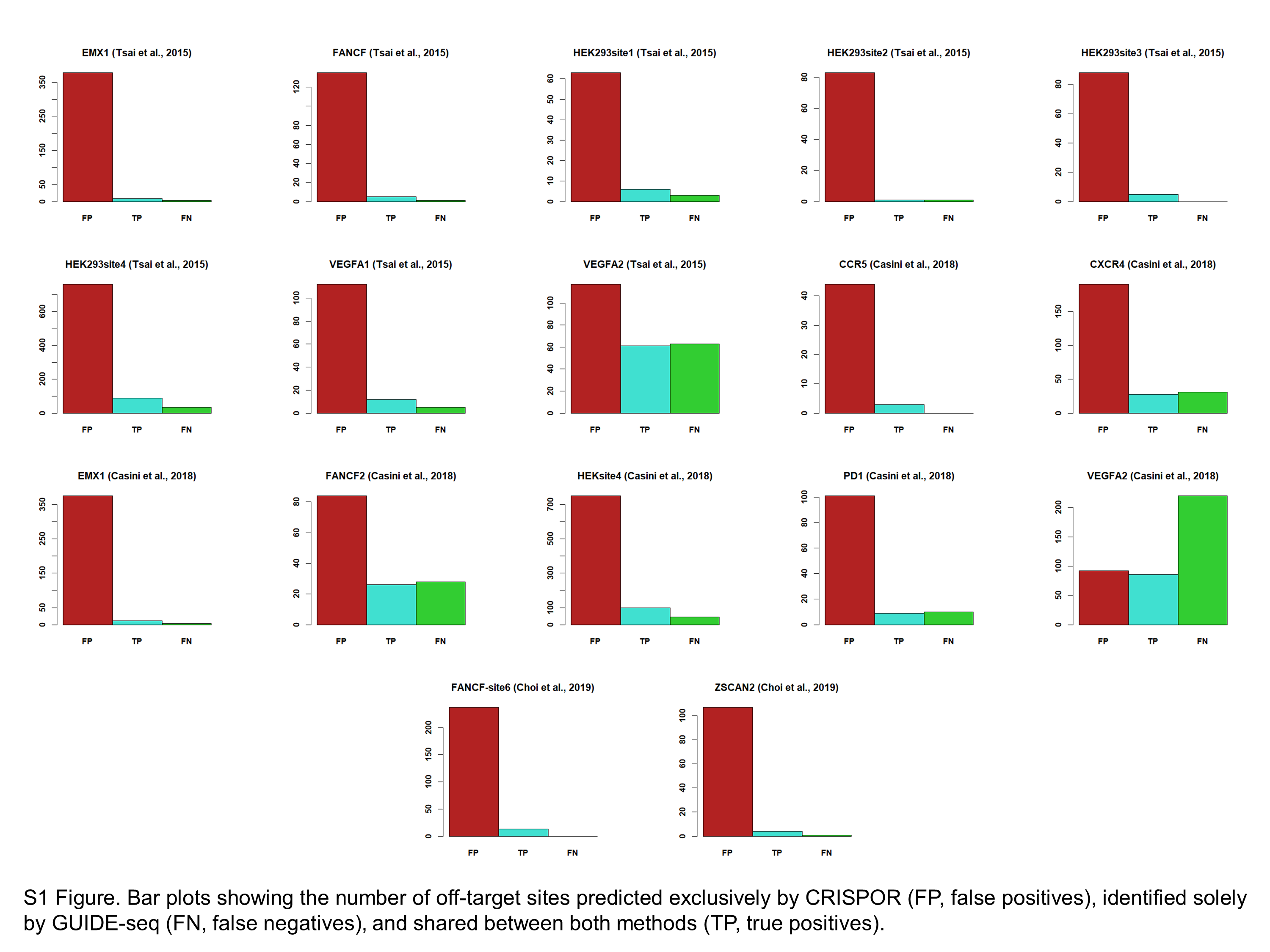

Supplement: S1 File — S1 Fig. Bar plots showing the number of off-target sites predicted exclusively by CRISPOR (FP, false positives), identified solely by GUIDE-seq (FN, false negatives), and shared between both methods (TP, true positives). S2 Fig. Distributions of CRISOT scores for the FP, TP, and FN groups. The mean and standard deviation for each group are shown. S3 Fig. Relationship between Spearman’s rank correlation coefficient and the Euclidean distance. Red dots indicate cases with statistically significant Spearman’s rank correlations. S4 Fig. Relationship between the Euclidean distance and the Pearson correlation coefficient. Red dots indicate cases with statistically significant Spearman’s rank correlations. S5 Fig. Spearman’s rank correlation coefficients for sequence patterns surrounding the VEGFA2 and VEGFA3 target sites across various Cas enzymes. All correlations were statistically significant with small EDs (<0.2). S6 Fig. Spearman’s rank correlation coefficients for sequence patterns surrounding the EMX1 and VEGFA3 target sites using WT SpCas9 across three different studies and cell lines. All correlations were statistically significant with small EDs (<0.2). (ZIP) [file pone.0328905.s001.zip › suppl_figures/S1_Fig.TIF]

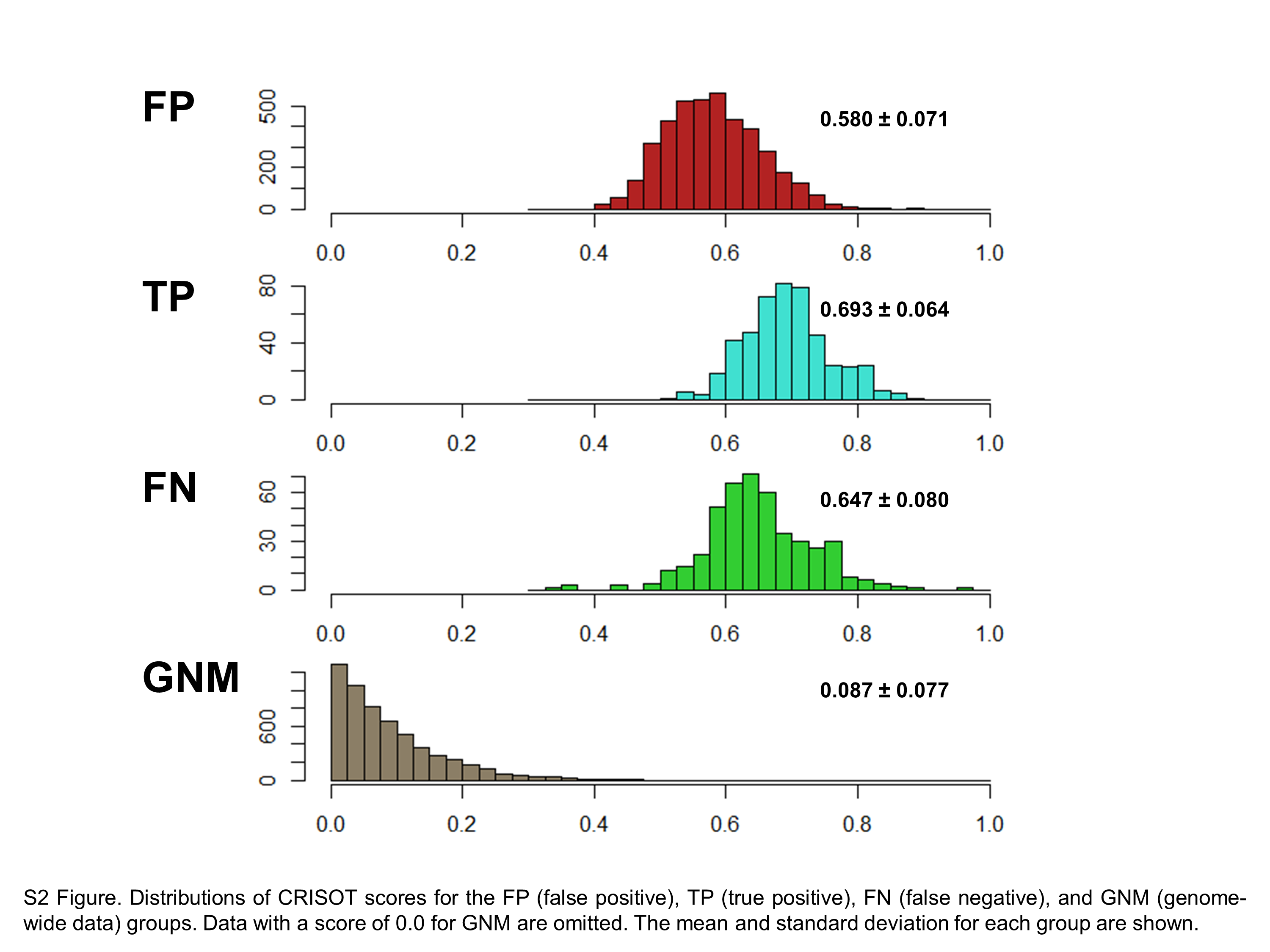

Supplement: S1 File — S1 Fig. Bar plots showing the number of off-target sites predicted exclusively by CRISPOR (FP, false positives), identified solely by GUIDE-seq (FN, false negatives), and shared between both methods (TP, true positives). S2 Fig. Distributions of CRISOT scores for the FP, TP, and FN groups. The mean and standard deviation for each group are shown. S3 Fig. Relationship between Spearman’s rank correlation coefficient and the Euclidean distance. Red dots indicate cases with statistically significant Spearman’s rank correlations. S4 Fig. Relationship between the Euclidean distance and the Pearson correlation coefficient. Red dots indicate cases with statistically significant Spearman’s rank correlations. S5 Fig. Spearman’s rank correlation coefficients for sequence patterns surrounding the VEGFA2 and VEGFA3 target sites across various Cas enzymes. All correlations were statistically significant with small EDs (<0.2). S6 Fig. Spearman’s rank correlation coefficients for sequence patterns surrounding the EMX1 and VEGFA3 target sites using WT SpCas9 across three different studies and cell lines. All correlations were statistically significant with small EDs (<0.2). (ZIP) [file pone.0328905.s001.zip › suppl_figures/S2_Fig.TIF]

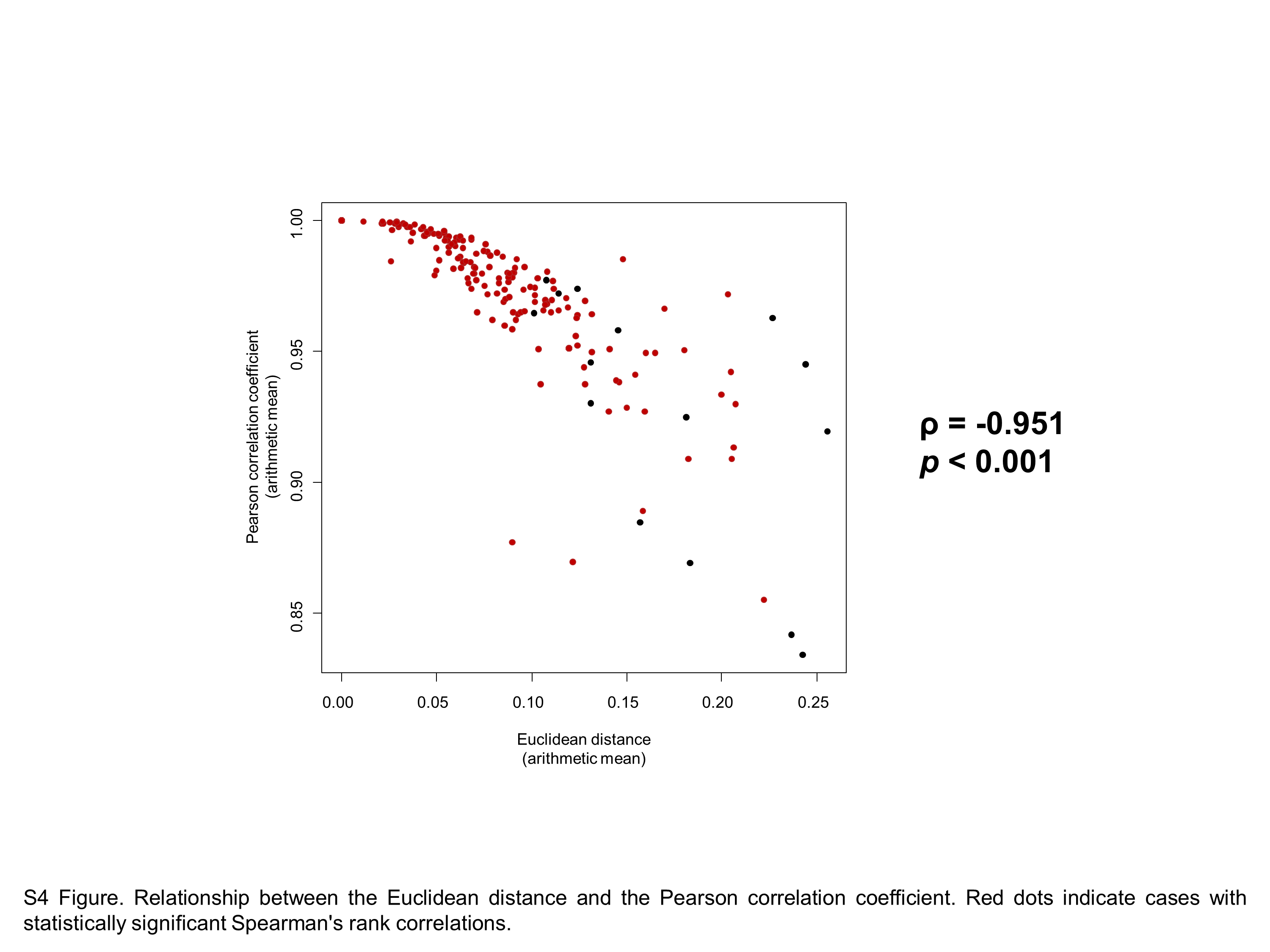

Supplement: S1 File — S1 Fig. Bar plots showing the number of off-target sites predicted exclusively by CRISPOR (FP, false positives), identified solely by GUIDE-seq (FN, false negatives), and shared between both methods (TP, true positives). S2 Fig. Distributions of CRISOT scores for the FP, TP, and FN groups. The mean and standard deviation for each group are shown. S3 Fig. Relationship between Spearman’s rank correlation coefficient and the Euclidean distance. Red dots indicate cases with statistically significant Spearman’s rank correlations. S4 Fig. Relationship between the Euclidean distance and the Pearson correlation coefficient. Red dots indicate cases with statistically significant Spearman’s rank correlations. S5 Fig. Spearman’s rank correlation coefficients for sequence patterns surrounding the VEGFA2 and VEGFA3 target sites across various Cas enzymes. All correlations were statistically significant with small EDs (<0.2). S6 Fig. Spearman’s rank correlation coefficients for sequence patterns surrounding the EMX1 and VEGFA3 target sites using WT SpCas9 across three different studies and cell lines. All correlations were statistically significant with small EDs (<0.2). (ZIP) [file pone.0328905.s001.zip › suppl_figures/S4_Fig.TIF]

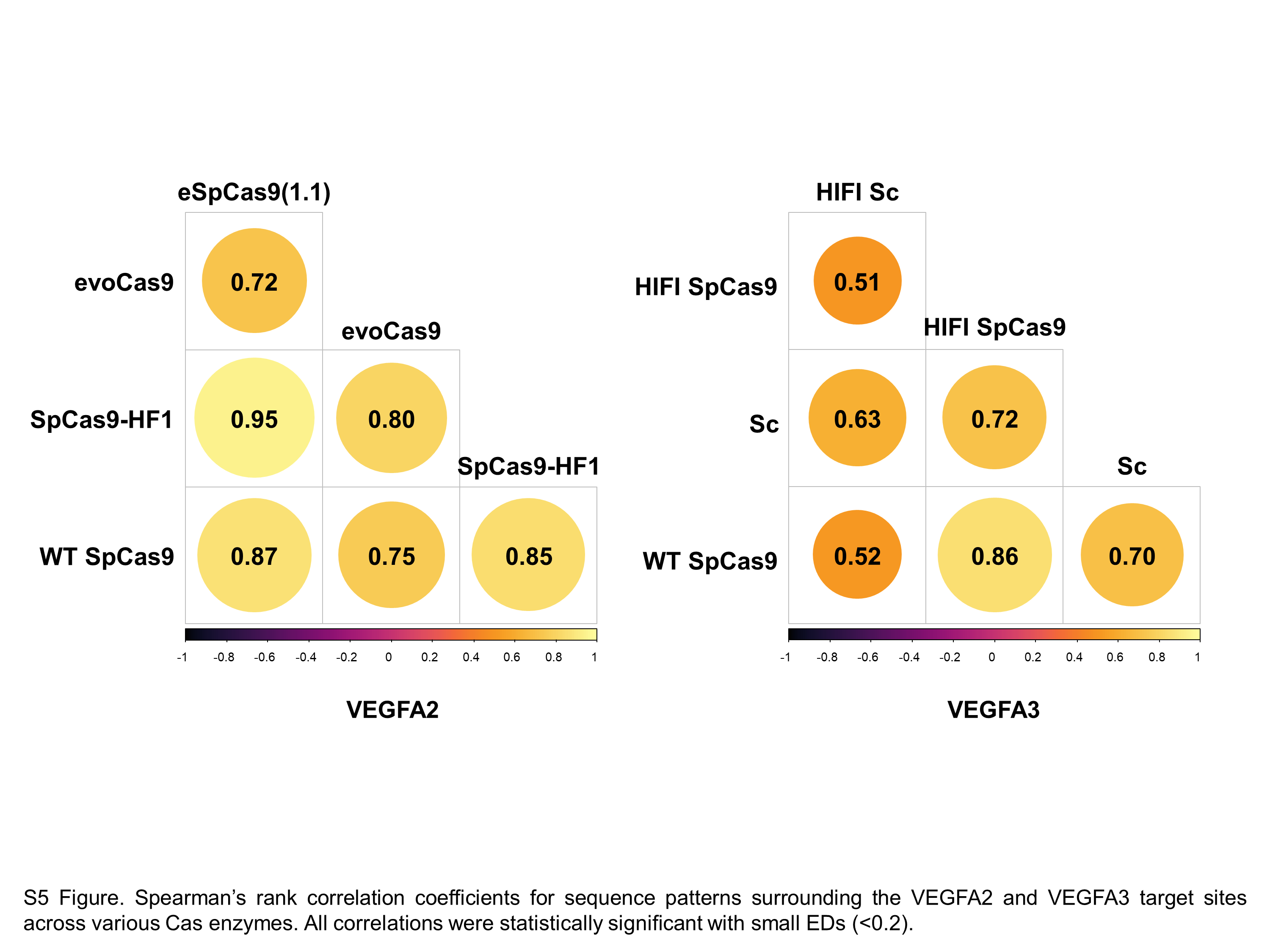

Supplement: S1 File — S1 Fig. Bar plots showing the number of off-target sites predicted exclusively by CRISPOR (FP, false positives), identified solely by GUIDE-seq (FN, false negatives), and shared between both methods (TP, true positives). S2 Fig. Distributions of CRISOT scores for the FP, TP, and FN groups. The mean and standard deviation for each group are shown. S3 Fig. Relationship between Spearman’s rank correlation coefficient and the Euclidean distance. Red dots indicate cases with statistically significant Spearman’s rank correlations. S4 Fig. Relationship between the Euclidean distance and the Pearson correlation coefficient. Red dots indicate cases with statistically significant Spearman’s rank correlations. S5 Fig. Spearman’s rank correlation coefficients for sequence patterns surrounding the VEGFA2 and VEGFA3 target sites across various Cas enzymes. All correlations were statistically significant with small EDs (<0.2). S6 Fig. Spearman’s rank correlation coefficients for sequence patterns surrounding the EMX1 and VEGFA3 target sites using WT SpCas9 across three different studies and cell lines. All correlations were statistically significant with small EDs (<0.2). (ZIP) [file pone.0328905.s001.zip › suppl_figures/S5_Fig.TIF]

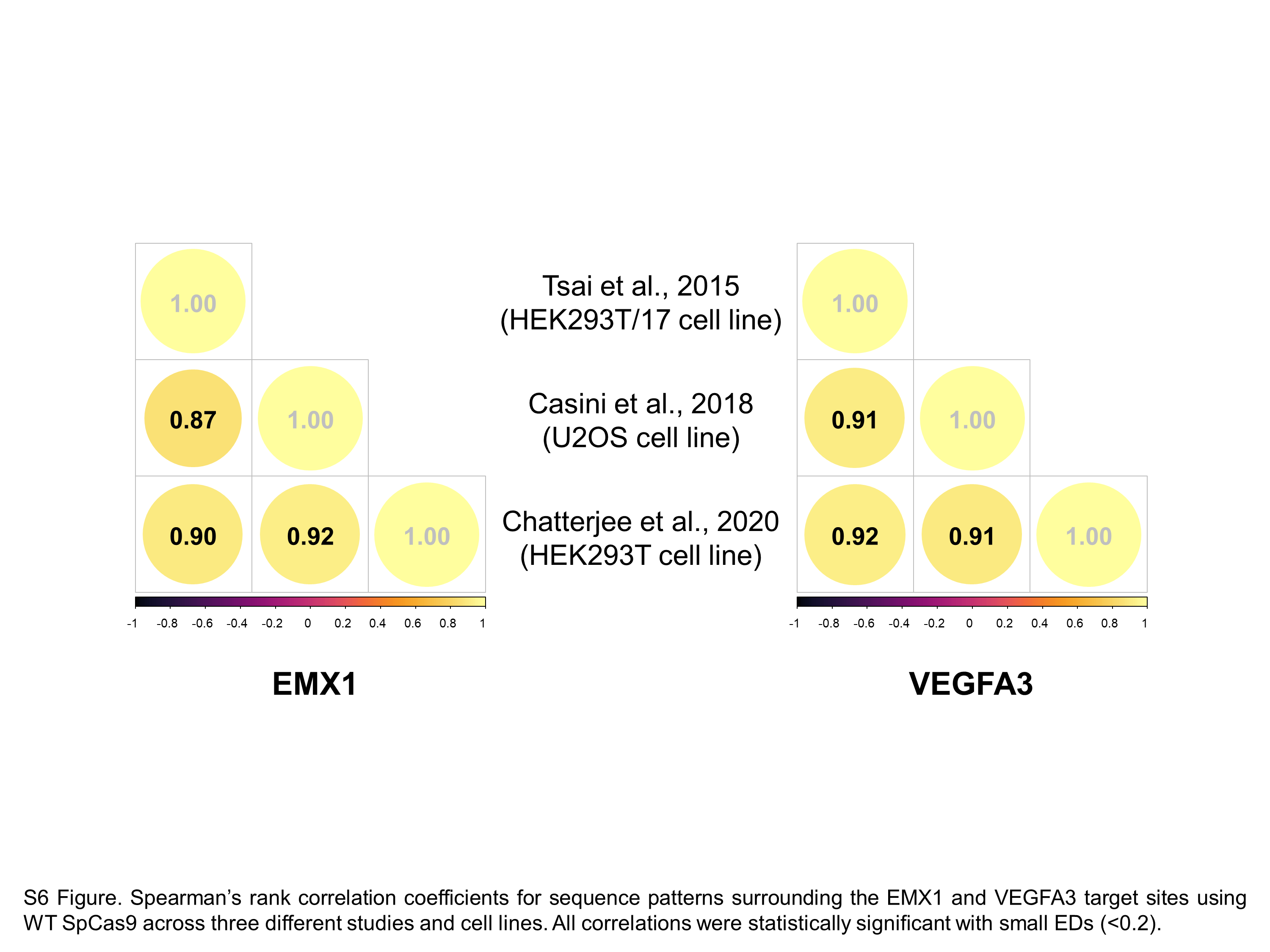

Supplement: S1 File — S1 Fig. Bar plots showing the number of off-target sites predicted exclusively by CRISPOR (FP, false positives), identified solely by GUIDE-seq (FN, false negatives), and shared between both methods (TP, true positives). S2 Fig. Distributions of CRISOT scores for the FP, TP, and FN groups. The mean and standard deviation for each group are shown. S3 Fig. Relationship between Spearman’s rank correlation coefficient and the Euclidean distance. Red dots indicate cases with statistically significant Spearman’s rank correlations. S4 Fig. Relationship between the Euclidean distance and the Pearson correlation coefficient. Red dots indicate cases with statistically significant Spearman’s rank correlations. S5 Fig. Spearman’s rank correlation coefficients for sequence patterns surrounding the VEGFA2 and VEGFA3 target sites across various Cas enzymes. All correlations were statistically significant with small EDs (<0.2). S6 Fig. Spearman’s rank correlation coefficients for sequence patterns surrounding the EMX1 and VEGFA3 target sites using WT SpCas9 across three different studies and cell lines. All correlations were statistically significant with small EDs (<0.2). (ZIP) [file pone.0328905.s001.zip › suppl_figures/S6_Fig.TIF]
