# Supplementary material for: Off-target sequence variations driven by the intrinsic properties of the Cas–sgRNA–DNA complex in genome editing
Source: PLoS One. 2025 Jul 18;20(7):e0328905. doi: 10.1371/journal.pone.0328905 (PMC12273960; doi:10.1371/journal.pone.0328905)
Supplement: S2 File — (ZIP) [file pone.0328905.s002.zip › suppl_tables/S1_Table.pdf]

S1 Table. Details of the GUIDE-seq datasets used in this study.

Study	Experimental Condition	Enzyme	Cell line	Target site
Tsai et al., 2015	HEK293 cells were transfected using Lonza Nucleofection with 300 ng pCAG-Cas9 + 150 ng gRNA + 5 pmol dsODN	WT SpCas9	HEK293	HEK293site4, HEK293site3, HEK293site2, HEK293site1,
	U2OS cells were transfected using Lonza Nucleofection with 500 ng pCAG-Cas9 + 250 ng gRNA + 100 pmol dsODN		U2OS	FANCF, EMX1, VEGFA3, VEGFA2, VEGFA1
Casini et al., 2018	2 × 105 293T/17 cells were transfected with 750 ng Cas9 plasmid + 250 ng sg-RNA + 10 pmol dsODN + 50 ng pEGFP-IRES-Puro plasmid	eSpCas9(1.1), evoCas9, SpCas9-HF1, WTSpCas9	HEK293T/17	EMX1, VEGFA2, FANCF2, HEKSITE4, PD1, CXCR4, CCR5, VEGFA3
Zhou et al., 2022	100 pmol dsODN tag was electroporated with Cas12a and gRNA expression plasmids using Neon Transfection System	Lb, LbK538R, As, AsK548R	U2OS	SITE1, POLQ2,
	100 pmol dsODN tag was transfected into HEK293T cells with Cas12a and gRNA expression plasmids (As desribed in previously Tsai et al., 2015)	Lb, LbK538R, As, AsK548R, Lb2, Lb2K518R	HEK293T	SITE1, POLQ1, POLQ2, PRKCH, B2M

Chatterjee et al., 2020	HEK293T cells were electroporated in a 24-well plate with 500 ng Cas9 + 500 ng sgRNA + 10ng mCherry plasmids + 7.5 pmol annealed GUIDE-seq oligonucleotide, using Neon nucleofection system (As described previously in Tsai et al., 2015)	Sc, HIFI_Sc, WT_SpyCas9, HIFI_SpyCas9	HEK293T	VEGFA3, EMX1
Tan et al., 2019	See Tsai et al., 2015	eSpCas9, HF1, SaCas9-HF, WTSaCas9, S-HF, SaCas9 variants	HEK293T	RUNX1_13, VEGFA_8, CCR5_1, CCR5_2, AAVS1_2, AAVS1_3, AAVS1_4, AAVS1_5, EMX1_sg3, EMX1_sg6, EMX1_sg7, EMX1_sg1, EMX1_sg2, EMX1_sg5, EMX1_6, FANCF_13
Choi et al., 2019	For each 1.5 million OVCAR8-ADR cells infected with SpCas9 variants and gRNAs were electroporated with 1,000 mol freshly annealed GUIDE-seq dsODN using 100 µl Neon tips	eSpCas9(1.1), evoCas9, HypaCas9, OptiHF-SpCas9, Opti-SpCas9, Sniper-Cas9, WT SpCas9	OVCAR8-ADR	FANCF6, ZSCAN2, EMX1_SITE_3
