# Supplementary material for: Off-target sequence variations driven by the intrinsic properties of the Cas–sgRNA–DNA complex in genome editing
Source: PLoS One. 2025 Jul 18;20(7):e0328905. doi: 10.1371/journal.pone.0328905 (PMC12273960; doi:10.1371/journal.pone.0328905)
Supplement: S2 File — (ZIP) [file pone.0328905.s002.zip › suppl_tables/S2_Table.pdf]

S2 Table. Accession numbers of the sequence data used in this study.

Study	Accession number	R1/R2	Sequence number (before trimming)	Sequence number (after trimming)	Enzyme	Target site	Target sequence
Tan et al. (2015)	SRR1695629	R1	2656678	911210	WT-SpCas9	VEGFA1	GGGTGGGGGGAGTTTGCTCCAGG
		R2	2656678	911210			
	SRR1695630	R1	2022010	871803	WT-SpCas9	VEGFA2	GACCCCCTCCACCCCGCCTCCGG
		R2	2022010	871803			
	SRR1695632	R1	2064846	845533	WT-SpCas9	VEGFA3	GGTGAGTGAGTGTGTGCGTGTGG
		R2	2064846	845533			
	SRR1695633	R1	3293444	1067450	WT-SpCas9	EMX1	GAGTCCGAGCAGAAGAAGAAGGG
		R2	3293444	1067450			
	SRR1695634	R1	2995443	957742	WT-SpCas9	FANCF	GGAATCCCTTCTGCAGCACCTGG
		R2	2995443	957742			
	SRR1695641	R1	1221479	818336	WT-SpCas9	HEK293site1	GGGAAAGACCCAGCATCCGTGGG
		R2	1221479	818336			
	SRR1695642	R1	1153766	775620	WT-SpCas9	HEK293site2	GAACACAAAGCATAGACTGCGGG
		R2	1153766	775620			
	SRR1695643	R1	1019643	669991	WT-SpCas9	HEK293site3	GGCCCAGACTGAGCACGTGATGG
		R2	1019643	669991			
	SRR1695644	R1	1286458	845631	WT-SpCas9	HEK293site4	GGCACTGCGGCTGGAGGTGGGGG
		R2	1286458	845631			
Casini et al. (2017)	SRR9651616	R1	2321144	1557301	eSpCas9(1.1)	EMX1	GAGTCCGAGCAGAAGAAGAAGGG
		R2	2321144	1557301			
	SRR9651617	R1	1992400	1460910	SpCas9-HF1	EMX1	GAGTCCGAGCAGAAGAAGAAGGG
		R2	1992400	1460910			
	SRR9651618	R1	2388296	1630061	evoCas9	HEKsite4	GGCACTGCGGCTGGAGGTGGGGG
		R2	2388296	1630061			
	SRR9651619	R1	2157985	1487154	WTSpCas9	HEKsite4	GGCACTGCGGCTGGAGGTGGGGG
		R2	2157985	1487154			
	SRR9651620	R1	2213646	1572535	eSpCas9(1.1)	FANCF2	GCTGCAGAAGGGATTCCATGAGG
		R2	2213646	1572535			
	SRR9651621	R1	2278117	1739595	SpCas9-HF1	FANCF2	GCTGCAGAAGGGATTCCATGAGG
		R2	2278117	1739595			
	SRR9651622	R1	2267816	1478833	evoCas9	EMX1	GAGTCCGAGCAGAAGAAGAAGGG
		R2	2267816	1478833			
	SRR9651623	R1	1815543	1139278	WTSpCas9	EMX1	GAGTCCGAGCAGAAGAAGAAGGG
		R2	1815543	1139278			
	SRR9651624	R1	1991187	1294831	eSpCas9(1.1)	HEKsite4	GGCACTGCGGCTGGAGGTGGGGG
		R2	1991187	1294831			
	SRR9651625	R1	1951020	1253632	SpCas9-HF1	HEKsite4	GGCACTGCGGCTGGAGGTGGGGG
		R2	1951020	1253632			
	SRR9651626	R1	3524255	2335446	SpCas9-HF1	VEGFA2	GACCCCCTCCACCCCGCCTCCGG
		R2	3524255	2335446			
	SRR9651627	R1	5200155	3315255	evoCas9	VEGFA2	GACCCCCTCCACCCCGCCTCCGG
		R2	5200155	3315255			
	SRR9651629	R1	2861275	2061850	eSpCas9(1.1)	VEGFA2	GACCCCCTCCACCCCGCCTCCGG
		R2	2861275	2061850			
	SRR9651633	R1	2211333	1497259	evoCas9	FANCF2	GCTGCAGAAGGGATTCCATGAGG
		R2	2211333	1497259			
	SRR9651634	R1	2056403	1392057	WTSpCas9	FANCF2	GCTGCAGAAGGGATTCCATGAGG
		R2	2056403	1392057			
	SRR9651667	R1	2826096	1975602	SpCas9-HF1	PD1	GGCCAGGATGGTTCTTAGGTAGG
		R2	2826096	1975602			
	SRR9651668	R1	2084547	1174992	eSpCas9(1.1)	PD1	GGCCAGGATGGTTCTTAGGTAGG
		R2	2084547	1174992			
	SRR9651669	R1	1895674	1229283	WTSpCas9	PD1	GGCCAGGATGGTTCTTAGGTAGG
		R2	1895674	1229283			
	SRR9651670	R1	2266621	1531155	evoCas9	PD1	GGCCAGGATGGTTCTTAGGTAGG
		R2	2266621	1531155			

	SRR9651671	R1	1796155	1383368 SpCas9-HF1	VEGFA3	GGTGAGTGAGTGTGTGCGTGNGG
		R2	1796155	1383368		
	SRR9651672	R1	2548823	1894875 eSpCas9(1.1)	VEGFA3	GGTGAGTGAGTGTGTGCGTGTGG
		R2	2548823	1894875		
	SRR9651673	R1	1920875	1315031 WTSpCas9	VEGFA3	GGTGAGTGAGTGTGTGCGTGTGG
		R2	1920875	1315031		
	SRR9651674	R1	2051103	1362382 evoCas9	VEGFA3	GGTGAGTGAGTGTGTGCGTGTGG
		R2	2051103	1362382		
	SRR9651675	R1	2389131	1655648 WTSpCas9	VEGFA3	GGTGAGTGAGTGTGTGCGTGTGG
		R2	2389131	1655648		
	SRR9651676	R1	5176078	2851485 WTSpCas9	VEGFA2	GACCCCCTCCACCCCGCCTCCGG
		R2	5176078	2851485		
	SRR9651687	R1	2011482	1540023 evoCas9	CCR5	GGTACCTATCGATTGTCAGGAGG
		R2	2011482	1540023		
	SRR9651688	R1	2108194	1577399 WTSpCas9	CCR5	GGTACCTATCGATTGTCAGGAGG
		R2	2108194	1577399		
	SRR9651689	R1	2506464	1871949 eSpCas9(1.1)	CCR5	GGTACCTATCGATTGTCAGGAGG
		R2	2506464	1871949		
	SRR9651690	R1	2750532	2112468 SpCas9-HF1	CCR5	GGTACCTATCGATTGTCAGGAGG
		R2	2750532	2112468		
	SRR9651691	R1	2527064	1750426 evoCas9	CXCR4	GAAGCGTGATGACAAAGAGGAGG
		R2	2527064	1750426		
	SRR9651692	R1	2255948	1701300 WTSpCas9	CXCR4	GAAGCGTGATGACAAAGAGGAGG
		R2	2255948	1701300		
	SRR9651693	R1	2161752	1618737 eSpCas9(1.1)	CXCR4	GAAGCGTGATGACAAAGAGGAGG
		R2	2161752	1618737		
	SRR9651695	R1	1769257	1233450 SpCas9-HF1	CXCR4	GAAGCGTGATGACAAAGAGGAGG
		R2	1769257	1233450		
Zhou et al. (2021)	SRR16229957	R1	2934983	1582358 As	B2M	TTTACTCACGTCATCCAGCAGAGAATG
		R2	2934983	1582358		
	SRR16229958	R1	3454746	1816664 AsK548R	B2M	TTTACTCACGTCATCCAGCAGAGAATG
		R2	3454746	1816664		
	SRR16229959	R1	3112798	1513541 AsK548R	POLQ1	TTTAGGCATGAATTATAATGCTGTTGG
		R2	3112798	1513541		
	SRR16229960	R1	5395747	1986923 AsK548R	POLQ2	TTTAAACAGAAACATGCATAAAACAAG
		R2	5395747	1986923		
	SRR16229961	R1	2978510	1394725 AsK548R	PRKCH	TTTGGGGACGGGGAGAAGGAAAAGAGG
		R2	2978510	1394725		
	SRR16229962	R1	4474948	1791088 AsK548R	Site 1	TTTCTGATGGTCCATACCTGTTACACT
		R2	4474948	1791088		
	SRR16229963	R1	6396592	2970485 As	POLQ1	TTTAGGCATGAATTATAATGCTGTTGG
		R2	6396592	2970485		
	SRR16229964	R1	5092420	2389846 As	POLQ2	TTTAAACAGAAACATGCATAAAACAAG
		R2	5092420	2389846		
	SRR16229965	R1	3169001	1497865 As	PRKCH	TTTGGGGACGGGGAGAAGGAAAAGAGG
		R2	3169001	1497865		
	SRR16229966	R1	3948340	2034773 As	Site 1	TTTCTGATGGTCCATACCTGTTACACT
		R2	3948340	2034773		
	SRR16229967	R1	3097765	678605 Lb2	B2M	TTTACTCACGTCATCCAGCAGAGAATG
		R2	3097765	678605		
	SRR16229968	R1	4311509	1665890 Lb2K518R	B2M	TTTACTCACGTCATCCAGCAGAGAATG
		R2	4311509	1665890		
	SRR16229969	R1	3272708	1672376 Lb2K518R	POLQ1	TTTAGGCATGAATTATAATGCTGTTGG
		R2	3272708	1672376		
	SRR16229970	R1	5845249	3192086 Lb2K518R	POLQ2	TTTAAACAGAAACATGCATAAAACAAG
		R2	5845249	3192086		
	SRR16229971	R1	3134575	1512412 Lb2K518R	PRKCH	TTTGGGGACGGGGAGAAGGAAAAGAGG
		R2	3134575	1512412		
	SRR16229972	R1	5098162	2083876 Lb2K518R	Site 1	TTTCTGATGGTCCATACCTGTTACACT
		R2	5098162	2083876		

	SRR16229973	R1	5717213	2325278	Lb2	POLQ1	TTTAGGCATGAATTATAATGCTGTTGG
		R2	5717213	2325278			
	SRR16229974	R1	5379030	2548715	Lb2	POLQ2	TTTAAACAGAAACATGCATAAAACAAG
		R2	5379030	2548715			
	SRR16229975	R1	6087204	2706307	Lb2	PRKCH	TTTGGGGACGGGGAGAAGGAAAAGAGG
		R2	6087204	2706307			
	SRR16229976	R1	3704044	1600279	Lb2	Site 1	TTTCTGATGGTCCATACCTGTTACACT
		R2	3704044	1600279			
	SRR16229977	R1	3811636	1811706	Lb	B2M	TTTACTCACGTCATCCAGCAGAGAATG
		R2	3811636	1811706			
	SRR16229978	R1	4029182	1972804	LbK538R	B2M	TTTACTCACGTCATCCAGCAGAGAATG
		R2	4029182	1972804			
	SRR16229979	R1	5058303	2147768	LbK538R	POLQ1	TTTAGGCATGAATTATAATGCTGTTGG
		R2	5058303	2147768			
	SRR16229980	R1	5383363	2733051	LbK538R	POLQ2	TTTAAACAGAAACATGCATAAAACAAG
		R2	5383363	2733051			
	SRR16229981	R1	2860181	1455709	LbK538R	PRKCH	TTTGGGGACGGGGAGAAGGAAAAGAGG
		R2	2860181	1455709			
	SRR16229982	R1	4411794	2412528	LbK538R	Site1	TTTCTGATGGTCCATACCTGTTACACT
		R2	4411794	2412528			
	SRR16229983	R1	3210333	1395897	Lb	POLQ1	TTTAGGCATGAATTATAATGCTGTTGG
		R2	3210333	1395897			
	SRR16229984	R1	6463067	3611670	Lb	POLQ2	TTTAAACAGAAACATGCATAAAACAAG
		R2	6463067	3611670			
	SRR16229985	R1	3523349	1808090	Lb	PRKCH	TTTGGGGACGGGGAGAAGGAAAAGAGG
		R2	3523349	1808090			
	SRR16229986	R1	4059579	1524722	Lb	Site 1	TTTCTGATGGTCCATACCTGTTACACT
		R2	4059579	1524722			
	SRR16229987	R1	6259535	2174654	AsK548R	POLQ2	TTTAAACAGAAACATGCATAAAACAAG
		R2	6259535	2174654			
	SRR16229988	R1	5595851	2359503	AsK548R	Site 1	TTTCTGATGGTCCATACCTGTTACACT
		R2	5595851	2359503			
	SRR16229989	R1	6725562	1908433	As	POLQ2	TTTAAACAGAAACATGCATAAAACAAG
		R2	6725562	1908433			
	SRR16229990	R1	7725990	2701224	As	Site 1	TTTCTGATGGTCCATACCTGTTACACT
		R2	7725990	2701224			
	SRR16229991	R1	7089410	2109079	LbK538R	Site 1	TTTCTGATGGTCCATACCTGTTACACT
		R2	7089410	2109079			
	SRR16229992	R1	6178660	2084796	LbK538R	POLQ2	TTTAAACAGAAACATGCATAAAACAAG
		R2	6178660	2084796			
	SRR16229993	R1	7782163	2673175	Lb	POLQ2	TTTAAACAGAAACATGCATAAAACAAG
		R2	7782163	2673175			
	SRR16229994	R1	9172527	4031647	Lb	Site 1	TTTCTGATGGTCCATACCTGTTACACT
		R2	9172527	4031647			
Chatterjee et al. (2020)	SRR11479175	R1	255673	205057	HIFI SpCas9	STS3 2	GGTGAGTGAGTGTGTGCGTGTGG
		R2	255673	205057			
	SRR11479176	R1	276554	222236	HIFI SpCas9	STS3 1	GGTGAGTGAGTGTGTGCGTGTGG
		R2	276554	222236			
	SRR11479177	R1	73410	41192	WT SpCas9	STS4 4	GAGTCCGAGCAGAAGAAGAAGGG
		R2	73410	41192			
	SRR11479178	R1	73567	40787	WT SpCas9	STS4 3	GAGTCCGAGCAGAAGAAGAAGGG
		R2	73567	40787			
	SRR11479179	R1	65342	37104	WT SpCas9	STS4 2	GAGTCCGAGCAGAAGAAGAAGGG
		R2	65342	37104			
	SRR11479180	R1	68106	37902	WT SpCas9	STS4 1	GAGTCCGAGCAGAAGAAGAAGGG
		R2	68106	37902			
	SRR11479181	R1	57345	33532	Sc	STS4 4	GAGTCCGAGCAGAAGAAGAAGGG
		R2	57345	33532			
	SRR11479182	R1	59040	33912	Sc	STS4 3	GAGTCCGAGCAGAAGAAGAAGGG
		R2	59040	33912			

SRR11479183	R1	52394	31005	Sc	STS4 2	GAGTCCGAGCAGAAGAAGAAGGG
	R2	52394	31005			
SRR11479184	R1	53485	31069	Sc	STS4 1	GAGTCCGAGCAGAAGAAGAAGGG
	R2	53485	31069			
SRR11479185	R1	180769	72499	HIFI SpCas9	STS4 4	GAGTCCGAGCAGAAGAAGAAGGG
	R2	180769	72499			
SRR11479186	R1	197411	78654	HIFI SpCas9	STS4 3	GAGTCCGAGCAGAAGAAGAAGGG
	R2	197411	78654			
SRR11479187	R1	307728	243032	HIFI Sc	STS3 4	GGTGAGTGAGTGTGTGCGTGTGG
	R2	307728	243032			
SRR11479188	R1	169844	65796	HIFI SpCas9	STS4 2	GAGTCCGAGCAGAAGAAGAAGGG
	R2	169844	65796			
SRR11479189	R1	184465	75572	HIFI SpCas9	STS4 1	GAGTCCGAGCAGAAGAAGAAGGG
	R2	184465	75572			
SRR11479190	R1	46555	18351	HIFI Sc	STS4 4	GAGTCCGAGCAGAAGAAGAAGGG
	R2	46555	18351			
SRR11479191	R1	49073	19246	HIFI Sc	STS4 3	GAGTCCGAGCAGAAGAAGAAGGG
	R2	49073	19246			
SRR11479192	R1	42658	16254	HIFI Sc	STS4 2	GAGTCCGAGCAGAAGAAGAAGGG
	R2	42658	16254			
SRR11479193	R1	47210	19036	HIFI Sc	STS4 1	GAGTCCGAGCAGAAGAAGAAGGG
	R2	47210	19036			
SRR11479198	R1	331082	261970	HIFI Sc	STS3 3	GGTGAGTGAGTGTGTGCGTGTGG
	R2	331082	261970			
SRR11479199	R1	82876	36040	WT SpCas9	STS4 4	GAGTCCGAGCAGAAGAAGAAGGG
	R2	82876	36040			
SRR11479200	R1	90473	38565	WT SpCas9	STS4 3	GAGTCCGAGCAGAAGAAGAAGGG
	R2	90473	38565			
SRR11479201	R1	73663	31209	WT SpCas9	STS4 2	GAGTCCGAGCAGAAGAAGAAGGG
	R2	73663	31209			
SRR11479202	R1	87833	38104	WT SpCas9	STS4 1	GAGTCCGAGCAGAAGAAGAAGGG
	R2	87833	38104			
SRR11479203	R1	28921	14004	Sc	STS4 4	GAGTCCGAGCAGAAGAAGAAGGG
	R2	28921	14004			
SRR11479204	R1	31246	14916	Sc	STS4 3	GAGTCCGAGCAGAAGAAGAAGGG
	R2	31246	14916			
SRR11479205	R1	26966	12653	Sc	STS4 2	GAGTCCGAGCAGAAGAAGAAGGG
	R2	26966	12653			
SRR11479206	R1	29735	14658	Sc	STS4 1	GAGTCCGAGCAGAAGAAGAAGGG
	R2	29735	14658			
SRR11479207	R1	43079	25387	HIFI SpCas9	STS4 4	GAGTCCGAGCAGAAGAAGAAGGG
	R2	43079	25387			
SRR11479208	R1	42015	24567	HIFI SpCas9	STS4 3	GAGTCCGAGCAGAAGAAGAAGGG
	R2	42015	24567			
SRR11479209	R1	267222	211671	HIFI Sc	STS3 2	GGTGAGTGAGTGTGTGCGTGTGG
	R2	267222	211671			
SRR11479210	R1	37546	22429	HIFI SpCas9	STS4 2	GAGTCCGAGCAGAAGAAGAAGGG
	R2	37546	22429			
SRR11479211	R1	39089	23003	HIFI SpCas9	STS4 1	GAGTCCGAGCAGAAGAAGAAGGG
	R2	39089	23003			
SRR11479212	R1	80276	46569	HIFI Sc	STS4 4	GAGTCCGAGCAGAAGAAGAAGGG
	R2	80276	46569			
SRR11479213	R1	77279	44720	HIFI Sc	STS4 3	GAGTCCGAGCAGAAGAAGAAGGG
	R2	77279	44720			
SRR11479214	R1	70573	41355	HIFI Sc	STS4 2	GAGTCCGAGCAGAAGAAGAAGGG
	R2	70573	41355			
SRR11479215	R1	70196	40745	HIFI Sc	STS4 1	GAGTCCGAGCAGAAGAAGAAGGG
	R2	70196	40745			
SRR11479220	R1	292034	231408	HIFI Sc	STS3 1	GGTGAGTGAGTGTGTGCGTGTGG
	R2	292034	231408			

	SRR11479221	R1	255069	190591	WT SpCas9	STS3 4	GGTGAGTGAGTGTGTGCGTGTGG
		R2	255069	190591			
	SRR11479222	R1	273446	204225	WT SpCas9	STS3 3	GGTGAGTGAGTGTGTGCGTGTGG
		R2	273446	204225			
	SRR11479223	R1	218218	164279	WT SpCas9	STS3 2	GGTGAGTGAGTGTGTGCGTGTGG
		R2	218218	164279			
	SRR11479224	R1	239212	179125	WT SpCas9	STS3 1	GGTGAGTGAGTGTGTGCGTGTGG
		R2	239212	179125			
	SRR11479225	R1	274892	197706	Sc	STS3 4	GGTGAGTGAGTGTGTGCGTGTGG
		R2	274892	197706			
	SRR11479226	R1	291216	211884	Sc	STS3 3	GGTGAGTGAGTGTGTGCGTGTGG
		R2	291216	211884			
	SRR11479227	R1	233182	169545	Sc	STS3 2	GGTGAGTGAGTGTGTGCGTGTGG
		R2	233182	169545			
	SRR11479228	R1	253960	184349	Sc	STS3 1	GGTGAGTGAGTGTGTGCGTGTGG
		R2	253960	184349			
	SRR11479229	R1	321671	235541	HIFI SpCas9	STS3 4	GGTGAGTGAGTGTGTGCGTGTGG
		R2	321671	235541			
	SRR11479230	R1	325320	235550	HIFI SpCas9	STS3 3	GGTGAGTGAGTGTGTGCGTGTGG
		R2	325320	235550			
	SRR11479232	R1	310726	225983	HIFI SpCas9	STS3 2	GGTGAGTGAGTGTGTGCGTGTGG
		R2	310726	225983			
	SRR11479233	R1	312201	227840	HIFI SpCas9	STS3 1	GGTGAGTGAGTGTGTGCGTGTGG
		R2	312201	227840			
	SRR11479234	R1	176283	114014	HIFI Sc	STS3 4	GGTGAGTGAGTGTGTGCGTGTGG
		R2	176283	114014			
	SRR11479235	R1	185794	118453	HIFI Sc	STS3 3	GGTGAGTGAGTGTGTGCGTGTGG
		R2	185794	118453			
	SRR11479236	R1	174328	112191	HIFI Sc	STS3 2	GGTGAGTGAGTGTGTGCGTGTGG
		R2	174328	112191			
	SRR11479237	R1	176315	113451	HIFI Sc	STS3 1	GGTGAGTGAGTGTGTGCGTGTGG
		R2	176315	113451			
	SRR11479243	R1	316214	203995	WT SpCas9	STS3 4	GGTGAGTGAGTGTGTGCGTGTGG
		R2	316214	203995			
	SRR11479244	R1	323096	207407	WT SpCas9	STS3 3	GGTGAGTGAGTGTGTGCGTGTGG
		R2	323096	207407			
	SRR11479245	R1	310294	199314	WT SpCas9	STS3 2	GGTGAGTGAGTGTGTGCGTGTGG
		R2	310294	199314			
	SRR11479246	R1	303025	197788	WT SpCas9	STS3 1	GGTGAGTGAGTGTGTGCGTGTGG
		R2	303025	197788			
	SRR11479247	R1	18156	9835	Sc	STS3 4	GGTGAGTGAGTGTGTGCGTGTGG
		R2	18156	9835			
	SRR11479248	R1	18169	9713	Sc	STS3 3	GGTGAGTGAGTGTGTGCGTGTGG
		R2	18169	9713			
	SRR11479249	R1	17827	9659	Sc	STS3 2	GGTGAGTGAGTGTGTGCGTGTGG
		R2	17827	9659			
	SRR11479250	R1	17271	9617	Sc	STS3 1	GGTGAGTGAGTGTGTGCGTGTGG
		R2	17271	9617			
	SRR11479251	R1	302353	241625	HIFI SpCas9	STS3 4	GGTGAGTGAGTGTGTGCGTGTGG
		R2	302353	241625			
	SRR11479252	R1	322675	259076	HIFI SpCas9	STS3 3	GGTGAGTGAGTGTGTGCGTGTGG
		R2	322675	259076			
Tan et al. (2019)	ERR3454713	R1	688171	646437	SaCas9-R245A/N413A.Guide	VEGFA 8	GGGTGAGTGAGTGTGTGCGTGTGGGGT
		R2	688171	646437			
	ERR3454714	R1	358940	329682	SaCas9-R245A/N413A/N419A.Guide	VEGFA 8	GGGTGAGTGAGTGTGTGCGTGTGGGGT
		R2	358940	329682			
	ERR3454715	R1	1343285	1208969	SaCas9-R245A/N413A/R654A.Guide	VEGFA 8	GGGTGAGTGAGTGTGTGCGTGTGGGGT
		R2	1343285	1208969			
	ERR3454716	R1	1182244	1091441	SaCas9-R245A/N419A.Guide	VEGFA 8	GGGTGAGTGAGTGTGTGCGTGTGGGGT
		R2	1182244	1091441			

ERR3454717	R1	1034962	930847	SaCas9-R245A/N419A/R654A.Guide	VEGFA 8	GGGTGAGTGAGTGTGTGCGTGTGGGGT
	R2	1034962	930847			
ERR3454718	R1	935544	872328	SaCas9-R245A/R654A.Guide	VEGFA 8	GGGTGAGTGAGTGTGTGCGTGTGGGGT
	R2	935544	872328			
ERR3454719	R1	1065574	986042	SaCas9-N413A/N419A.Guide	VEGFA 8	GGGTGAGTGAGTGTGTGCGTGTGGGGT
	R2	1065574	986042			
ERR3454720	R1	1390410	1295336	SaCas9-N413A/N419A/R654A.Guide	VEGFA 8	GGGTGAGTGAGTGTGTGCGTGTGGGGT
	R2	1390410	1295336			
ERR3454721	R1	1001441	916341	SaCas9-N413A/R654A.Guide	VEGFA 8	GGGTGAGTGAGTGTGTGCGTGTGGGGT
	R2	1001441	916341			
ERR3454722	R1	424040	393136	SaCas9-N419A/R654A.Guide	VEGFA 8	GGGTGAGTGAGTGTGTGCGTGTGGGGT
	R2	424040	393136			
ERR3454723	R1	358560	329837	SaCas9-R245A/N413A.Guide	EMX1 6	GCAACCACAAACCCACGAGGGCAGAGT
	R2	358560	329837			
ERR3454724	R1	258618	232847	SaCas9-R245A/N413A/N419A.Guide	EMX1 6	GCAACCACAAACCCACGAGGGCAGAGT
	R2	258618	232847			
ERR3454725	R1	124534	118341	SaCas9-R245A/N413A/R654A.Guide	EMX1 6	GCAACCACAAACCCACGAGGGCAGAGT
	R2	124534	118341			
ERR3454726	R1	851390	783366	SaCas9-R245A/N419A.Guide	EMX1 6	GCAACCACAAACCCACGAGGGCAGAGT
	R2	851390	783366			
ERR3454727	R1	846671	782509	SaCas9-R245A/N419A/R654A.Guide	EMX1 6	GCAACCACAAACCCACGAGGGCAGAGT
	R2	846671	782509			
ERR3454728	R1	631448	539495	SaCas9-R245A/R654A.Guide	EMX1 6	GCAACCACAAACCCACGAGGGCAGAGT
	R2	631448	539495			
ERR3454729	R1	733383	623058	SaCas9-N413A/N419A.Guide	EMX1 6	GCAACCACAAACCCACGAGGGCAGAGT
	R2	733383	623058			
ERR3454730	R1	233316	198423	SaCas9-N413A/N419A/R654A.Guide	EMX1 6	GCAACCACAAACCCACGAGGGCAGAGT
	R2	233316	198423			
ERR3454731	R1	84253	66875	SaCas9-N413A/R654A.Guide	EMX1 6	GCAACCACAAACCCACGAGGGCAGAGT
	R2	84253	66875			
ERR3454732	R1	888878	794620	SaCas9-N419A/R654A.Guide	EMX1 6	GCAACCACAAACCCACGAGGGCAGAGT
	R2	888878	794620			
ERR3454733	R1	349446	302638	SaCas9-R245A/N413A.Guide	FANCF 13	GCAAGGCCCGGCGCACGGTGGCGGGGT
	R2	349446	302638			
ERR3454734	R1	581113	520519	SaCas9-R245A/N413A/N419A.Guide	FANCF 13	GCAAGGCCCGGCGCACGGTGGCGGGGT
	R2	581113	520519			
ERR3454735	R1	72379	68722	SaCas9-R245A/N413A/R654A.Guide	FANCF 13	GCAAGGCCCGGCGCACGGTGGCGGGGT
	R2	72379	68722			
ERR3454736	R1	317518	279783	SaCas9-R245A/N419A.Guide	FANCF 13	GCAAGGCCCGGCGCACGGTGGCGGGGT
	R2	317518	279783			
ERR3454737	R1	422667	350256	SaCas9-R245A/N419A/R654A.Guide	FANCF 13	GCAAGGCCCGGCGCACGGTGGCGGGGT
	R2	422667	350256			
ERR3454738	R1	320855	250096	SaCas9-R245A/R654A.Guide	FANCF 13	GCAAGGCCCGGCGCACGGTGGCGGGGT
	R2	320855	250096			
ERR3454739	R1	224435	167336	SaCas9-N413A/N419A.Guide	FANCF 13	GCAAGGCCCGGCGCACGGTGGCGGGGT
	R2	224435	167336			
ERR3454740	R1	413000	352770	SaCas9-N413A/N419A/R654A.Guide	FANCF 13	GCAAGGCCCGGCGCACGGTGGCGGGGT
	R2	413000	352770			
ERR3454741	R1	573087	527342	SaCas9-N413A/R654A.Guide	FANCF 13	GCAAGGCCCGGCGCACGGTGGCGGGGT
	R2	573087	527342			
ERR3454742	R1	1471755	1373128	SaCas9-N419A/R654A.Guide	FANCF 13	GCAAGGCCCGGCGCACGGTGGCGGGGT
	R2	1471755	1373128			
ERR3454743	R1	1107583	967714	WTSaCas9.Guide	VEGFA 3	GAGAGGGACACACAGATCTATTGGAAT
	R2	1107583	967714			
ERR3454744	R1	1059849	923543	WTSaCas9.Guide	CCR5 1	GATGTAGTCAGAGTGAAATGGCCGGGT
	R2	1059849	923543			
ERR3454745	R1	941583	783022	WTSaCas9.Guide	CCR5 2	GTTGCCCTAAGGATTAAATGAATGAAT
	R2	941583	783022			
ERR3454746	R1	959424	864689	WTSaCas9.Guide	AAVS1 2	GAGAGATGGCTCCAGGAAATGGGGGT
	R2	959424	864689			

ERR3454747	R1	850504	703002	WTSaCas9.Guide	AAVS1 3	GAGCCACATTAACCGGCCCTGGGAAT
	R2	850504	703002			
ERR3454748	R1	942455	849496	WTSaCas9.Guide	AAVS1 4	GACTAGGAAGGAGGAGGCCTAAGGAT
	R2	942455	849496			
ERR3454749	R1	1058542	950881	WTSaCas9.Guide	AAVS1 5	GAATCTGCCTAACAGGAGGTGGGGGT
	R2	1058542	950881			
ERR3454750	R1	396272	333850	WTSaCas9.Guide	EMX1 sg5	GCAAGCAGCACTCTGCCCTCGTGGGT
	R2	396272	333850			
ERR3454751	R1	1825633	1624893	WTSaCas9.Guide	EMX1 sg6	GCCTCCCCAAAGCCTGGCCAGGGAGT
	R2	1825633	1624893			
ERR3454752	R1	327199	287853	eSpCas9.Guide	RUNX1 13	GAAAGAGAGATGTAGGGCTAGAGG
	R2	327199	287853			
ERR3454753	R1	966168	799428	HyPa.Guide	RUNX1 13	GAAAGAGAGATGTAGGGCTAGAGG
	R2	966168	799428			
ERR3454754	R1	657986	547630	SaCas9-HF.Guide	CCR5 1	GATGTAGTCAGAGTGAAATGGCCGGGT
	R2	657986	547630			
ERR3454755	R1	445872	401310	SaCas9-HF.Guide	CCR5 2	GTTGCCCTAAGGATTAAATGAATGAAT
	R2	445872	401310			
ERR3454756	R1	895604	822755	SaCas9-HF.Guide	AAVS1 2	GAGAGATGGCTCCAGGAAATGGGGGT
	R2	895604	822755			
ERR3454757	R1	1039926	956251	SaCas9-HF.Guide	AAVS1 3	GAGCCACATTAACCGGCCCTGGGAAT
	R2	1039926	956251			
ERR3454758	R1	780656	697187	SaCas9-HF.Guide	AAVS1 4	GACTAGGAAGGAGGAGGCCTAAGGAT
	R2	780656	697187			
ERR3454759	R1	563580	430416	SaCas9-HF.Guide	AAVS1 5	GAATCTGCCTAACAGGAGGTGGGGGT
	R2	563580	430416			
ERR3454760	R1	905507	826574	SaCas9-HF.Guide	EMX1 sg3	GTGGCTGCTCTGGGGGCCTCCTGAGT
	R2	905507	826574			
ERR3454761	R1	1224440	1101934	SaCas9-HF.Guide	EMX1 sg6	GCCTCCCCAAAGCCTGGCCAGGGAGT
	R2	1224440	1101934			
ERR3454762	R1	759474	685788	HyPa.Guide	VEGFA 8	GGGTGAGTGAGTGTGTGCGTGTGG
	R2	759474	685788			
ERR3454764	R1	1722862	1704050	WTSaCas9.Guide	EMX1 sg2	TGGCCAGGCTTTGGGGAGGCCTGGAGT
	R2	1722862	1704050			
ERR3454765	R1	1895334	1872599	WTSaCas9.Guide	EMX1 sg3	GTGGCTGCTCTGGGGGCCTCCTGAGT
	R2	1895334	1872599			
ERR3454766	R1	1851044	1830711	SaCas9-HF.Guide	EMX1 sg7	GGCCAGGCTTTGGGGAGGCCTGGAGT
	R2	1851044	1830711			
ERR3454767	R1	1433688	1416982	S-HF.Guide	FANCF 13	GCAAGGCCCGGCGCACGGTGGCGGGGT
	R2	1433688	1416982			
ERR3454768	R1	1522930	1504661	S-HF.GUide	EMX1 sg6	GCCTCCCCAAAGCCTGGCCAGGGAGT
	R2	1522930	1504661			
ERR3454769	R1	1853325	1831190	S-HF.GUide	VEGFA 8	GGGTGAGTGAGTGTGTGCGTGTGGGGT
	R2	1853325	1831190			
ERR3454773	R1	1850293	1807951	SaCas9-HF.GUide	EMX1 sg1	GGCCTCCCCAAACGGTGGCCAGGGAGT
	R2	1850293	1807951			
ERR3454774	R1	2334934	2286365	SaCas9-HF.GUide	EMX1 sg2	TGGCCAGGCTTTGGGGAGGCCTGGAGT
	R2	2334934	2286365			
ERR3454776	R1	2031714	1988166	SaCas9-HF.GUide	EMX1 sg5	GCAAGCAGCACTCTGCCCTCGTGGGT
	R2	2031714	1988166			
ERR3454777	R1	2368927	2320852	WTSaCas9.GUide	EMX1 sg7	GGCCAGGCTTTGGGGAGGCCTGGAGT
	R2	2368927	2320852			
ERR3454778	R1	1995011	1946412	WTSaCas9.GUide	EMX1 sg1	GGCCTCCCCAAACGGTGGCCAGGGAGT
	R2	1995011	1946412			
ERR3454780	R1	2802679	2734596	HF1.Guide	RUNX1 13	GAAAGAGAGATGTAGGGCTAGAGG
	R2	2802679	2734596			
ERR3454781	R1	1370736	1295711	HF1.Guide	VEGFA 8	GGGTGAGTGAGTGTGTGCGTGTGG
	R2	1370736	1295711			
ERR3454782	R1	1608993	1572814	eSpCas9.Guide	VEGFA 8	GGGTGAGTGAGTGTGTGCGTGTGG
	R2	1608993	1572814			

Choi et al. (2019)	ERR3319813	R1	583717	578628	Sniper-Cas9	FANCF-site6 with an additional 5'G	GCTTGAGACCGCCAGAAGCTCGG
		R2	583717	578628			
	ERR3319814	R1	189720	187874	OptiHF-SpCas9	FANCF-site6 with an additional 5'G	GCTTGAGACCGCCAGAAGCTCGG
		R2	189720	187874			
	ERR3319815	R1	607299	600595	Sniper-Cas9	EMX1-site3 with an additional 5'G	GAGTCCGAGCAGAAGAAGAAGGG
		R2	607299	600595			
	ERR3319816	R1	80531	79630	OptiHF-SpCas9	EMX1-site3 with an additional 5'G	GAGTCCGAGCAGAAGAAGAAGGG
		R2	80531	79630			
	ERR3319817	R1	567800	561643	WT-SpCas9	ZSCAN2 with an additional 5'G	GTGCGGCAAGAGCTTCAGCCGGG
		R2	567800	561643			
	ERR3319818	R1	274442	271155	Opti-SpCas9	ZSCAN2 with an additional 5'G	GTGCGGCAAGAGCTTCAGCCGGG
		R2	274442	271155			
	ERR3319819	R1	567601	560662	Sniper-Cas9	ZSCAN2 with an additional 5'G	GTGCGGCAAGAGCTTCAGCCGGG
		R2	567601	560662			
	ERR3319820	R1	557747	552259	OptiHF-SpCas9	ZSCAN2 with an additional 5'G	GTGCGGCAAGAGCTTCAGCCGGG
		R2	557747	552259			
	ERR3319821	R1	4825	4775	WT SpCas9	FANCF-site6 with a starting 5'G	GCTTGAGACCGCCAGAAGCTCGG
		R2	4825	4775			
	ERR3319822	R1	30898	30761	Opti-SpCas9	FANCF-site6 with a starting 5'G	GCTTGAGACCGCCAGAAGCTCGG
		R2	30898	30761			
	ERR3319823	R1	96227	95159	eSpCas9(1.1)	FANCF-site6 with a starting 5'G	GCTTGAGACCGCCAGAAGCTCGG
		R2	96227	95159			
	ERR3319824	R1	344506	340862	HypaCas9	FANCF-site6 with a starting 5'G	GCTTGAGACCGCCAGAAGCTCGG
		R2	344506	340862			
	ERR3319825	R1	893227	883011	evoCas9	FANCF-site6 with a starting 5'G	GCTTGAGACCGCCAGAAGCTCGG
		R2	893227	883011			
	ERR3319826	R1	542986	537395	Sniper-Cas9	FANCF-site6 with a starting 5'G	GCTTGAGACCGCCAGAAGCTCGG
		R2	542986	537395			
	ERR3319827	R1	493775	488322	OptiHF-SpCas9	FANCF-site6 with a starting 5'G	GCTTGAGACCGCCAGAAGCTCGG
		R2	493775	488322			
	ERR3319828	R1	326497	323473	WT SpCas9	ZSCAN2 with a starting 5'G	GTGCGGCAAGAGCTTCAGCCGGG
		R2	326497	323473			
	ERR3319829	R1	634323	627364	Opti-SpCas9	ZSCAN2 with a starting 5'G	GTGCGGCAAGAGCTTCAGCCGGG
		R2	634323	627364			
	ERR3319830	R1	666572	658801	eSpCas9(1.1)	ZSCAN2 with a starting 5'G	GTGCGGCAAGAGCTTCAGCCGGG
		R2	666572	658801			
	ERR3319831	R1	542926	535876	HypaCas9	ZSCAN2 with a starting 5'G	GTGCGGCAAGAGCTTCAGCCGGG
		R2	542926	535876			
	ERR3319832	R1	1440241	1425548	evoCas9	ZSCAN2 with a starting 5'G	GTGCGGCAAGAGCTTCAGCCGGG
		R2	1440241	1425548			
	ERR3319833	R1	603830	597652	Sniper-Cas9	ZSCAN2 with a starting 5'G	GTGCGGCAAGAGCTTCAGCCGGG
		R2	603830	597652			
	ERR3319834	R1	1015431	1004537	OptiHF-SpCas9	ZSCAN2 with a starting 5'G	GTGCGGCAAGAGCTTCAGCCGGG
		R2	1015431	1004537			
	ERR3319809	R1	374878	369283	WT-SpCas9	FANCF-site6 with an additional 5'G	GCTTGAGACCGCCAGAAGCTCGG
		R2	374878	369283			
	ERR3319810	R1	219360	215453	WT-SpCas9	EMX1-site3 with an additional 5'G	GAGTCCGAGCAGAAGAAGAAGGG
		R2	219360	215453			
	ERR3319811	R1	590243	582127	Opti-SpCas9	FANCF-site6 with an additional 5'G	GCTTGAGACCGCCAGAAGCTCGG
		R2	590243	582127			
	ERR3319812	R1	376515	371476	Opti-SpCas9	EMX1-site3 with an additional 5'G	GAGTCCGAGCAGAAGAAGAAGGG
		R2	376515	371476			
