# Supplementary material for: Off-target sequence variations driven by the intrinsic properties of the Cas–sgRNA–DNA complex in genome editing
Source: PLoS One. 2025 Jul 18;20(7):e0328905. doi: 10.1371/journal.pone.0328905 (PMC12273960; doi:10.1371/journal.pone.0328905)
Supplement: S2 File — (ZIP) [file pone.0328905.s002.zip › suppl_tables/S3_Table.pdf]

S3 Table. Recall and precision values for different target sites.

Target Site	Recall	Precision
VEGFA2 (Casini et al., 2018)	0.281	0.483
HEK293site2 (Tsai et al., 2015)	0.500	0.012
CXCR4 (Casini et al., 2018)	0.475	0.128
FANCF2 (Casini et al., 2018)	0.481	0.236
PD1 (Casini et al., 2018)	0.474	0.082
VEGFA2 (Tsai et al., 2015)	0.492	0.343
HEK293site1 (Tsai et al., 2015)	0.667	0.087
EMX1 (Casini et al., 2018)	0.692	0.023
HEKsite4 (Casini et al., 2018)	0.685	0.115
EMX1 (Tsai et al., 2015)	0.750	0.023
VEGFA1 (Tsai et al., 2015)	0.706	0.097
FANCF (Tsai et al., 2015)	0.833	0.036
HEK293site4 (Tsai et al., 2015)	0.732	0.106
EMX1 (Casini et al., 2018)	0.786	0.028
CCR5 (Casini et al., 2018)	1.000	0.064
ZSCAN2 (Choi et al., 2019)	0.800	0.036
HEK293site3 (Tsai et al., 2015)	1.000	0.054
FANCF-site6 (Choi et al., 2019)	1.000	0.052
