# Supplementary material for: Off-target sequence variations driven by the intrinsic properties of the Cas–sgRNA–DNA complex in genome editing
Source: PLoS One. 2025 Jul 18;20(7):e0328905. doi: 10.1371/journal.pone.0328905 (PMC12273960; doi:10.1371/journal.pone.0328905)
Supplement: S2 File — (ZIP) [file pone.0328905.s002.zip › suppl_tables/S4_Table.pdf]

S4 Table. Divergent and semidivergent target sequence patterns.

Class	Enzyme 1	Enzyme 2	Target site	Rank correlation ρ	Rank correlation *p*-value	Euclidean distance	Study
Divergent (*p* ≥ 0.05 for SRCC and ED ≥ 0.20)	AsK548R	Lb2	SITE1	0.07585	0.70689	0.24256	Zhou et al. 2021
	WTSpCas9	SpCas9HF1	HEKsite4	0.19462	0.37353	0.24396	Casini et al. 2017
	Lb2	Lb	SITE1	0.17908	0.37146	0.23666	Zhou et al. 2021
	evoCas9	WTSpCas9	FANCF2	0.39575	0.06159	0.22657	Casini et al. 2017
	WTSpCas9	eSpCas9	CXCR4	0.39774	0.06018	0.25545	Casini et al. 2017
Semi-divergent (*p* ≥ 0.05 for SRCC and ED < 0.20)	As	LbK538R	POLQ1	-0.10410	0.60536	0.13095	Zhou et al. 2021
	eSpCas9	SpCas9HF1	HEKsite4	0.25718	0.23616	0.18120	Casini et al. 2017
	Lb2K518R	Lb2	SITE1	0.26364	0.18394	0.18332	Zhou et al. 2021
	As	Lb2	POLQ1	0.26991	0.17333	0.13095	Zhou et al. 2021
	LbK538R	Lb	POLQ1	0.27064	0.17213	0.11434	Zhou et al. 2021
	AsK548R	Lb	POLQ1	0.29572	0.13423	0.12393	Zhou et al. 2021
	evoCas9	WTSpCas9	CCR5	0.32233	0.13361	0.10760	Casini et al. 2017
	WTSpCas9	SpCas9HF1	CCR5	0.32233	0.13361	0.10760	Casini et al. 2017
	AsK548R	LbK538R	POLQ1	0.32954	0.09323	0.10125	Zhou et al. 2021
	eSpCas9	evoCas9	FANCF2	0.36003	0.09151	0.14548	Casini et al. 2017
	Lb2	LbK538R	SITE1	0.33798	0.08466	0.15713	Zhou et al. 2021
Semi-divergent (*p* < 0.05 for SRCC and ED ≥ 0.20)	HIFI_Sc	WTSpCas9	EMX1	0.42673	0.04229	0.20722	Chatterjee et al. 2020
	Sc	WTSpCas9	EMX1	0.43148	0.03981	0.20005	Chatterjee et al. 2020
	As	Lb2	SITE1	0.40935	0.03398	0.22220	Zhou et al. 2021
	As	Lb2K518R	PRKCH	0.43230	0.02432	0.20655	Zhou et al. 2021
	WTSpCas9	eSpCas9	HEKsite4	0.53137	0.00907	0.20311	Casini et al. 2017
	Lb2K518R	Lb	PRKCH	0.50877	0.00673	0.20535	Zhou et al. 2021
	eSpCas9	WTSpCas9	FANCF2	0.55686	0.00578	0.20472	Casini et al. 2017
