# Supplementary material for: Off-target sequence variations driven by the intrinsic properties of the Cas–sgRNA–DNA complex in genome editing
Source: PLoS One. 2025 Jul 18;20(7):e0328905. doi: 10.1371/journal.pone.0328905 (PMC12273960; doi:10.1371/journal.pone.0328905)
Supplement: S2 File — (ZIP) [file pone.0328905.s002.zip › suppl_tables/S5_Table.pdf]

S5 Table. Similarity of target patterns for POLQ2 between the HEK293T and U2OS cell lines.

Enzyme	ρ	*p*	ED
As	0.713	<0.001	0.075
AsK548R	0.807	<0.001	0.062
Lb	0.882	<0.001	0.051
LbK538R	0.892	<0.001	0.026
