# Supplementary material for: Off-target sequence variations driven by the intrinsic properties of the Cas–sgRNA–DNA complex in genome editing
Source: PLoS One. 2025 Jul 18;20(7):e0328905. doi: 10.1371/journal.pone.0328905 (PMC12273960; doi:10.1371/journal.pone.0328905)
Supplement: Attachment — Submitted filename: review_Kurniawan_PlosOne_2024.pdf [file pone.0328905.s003.pdf]

The authors of the manuscript under review with the title "Off-target sequence variations driven by the intrinsic properties of the Cas-sgRNA-DNA complex in genome editing" evaluate the performance for off-target site predictions with the program CRISPOR on six independent GUIDE-seq datasets in genome editing applications. They furthermore compare off-target site sequences and find specific sequence motifs, which seem to be dependent on the used enzyme, cell-lines and other factors. Off-target site cleavages must be prohibited for many applications to limit genome editing only to the intended sites for safe use in the future.

The general research question is of great relevance and the principle approach to assess the benchmark between *in silico* predictions with *in vivo* or *in vitro* derived high throughput sequencing data is convincing.

However, I do have three major concerns with the current status of the manuscript:

a) The authors report about their extensive literature research, which unfortunately resulted in only 6 studies that made their sequencing data publicly available. It would be great to base the assessment on a more diverse data foundation, but I don't see any means how the authors should achieve that other than repeating the literature research, which was done back in 2022. Chances are high that further GUIDE-seq data have been submitted in between.

   On the other hand, the authors limit themselves to only ONE computational prediction tool, namely CRISPOR, although they state that "numerous *in silico* prediction tools have been developed" (line 82). Alternatives to CRISPOR are not even discussed in the manuscript. I expect the authors to re-do their analysis also for those other software - which should be well within their reach.

b) The authors did align off-target site sequences and quantified the alignment pattern by relative entropy. They formulate the background assumption that "equal frequency was assumed for the four nucleotides, setting p_g = 0.25" (line 182).
   I disagree with this assumption! If I am not mistaken, you design one target nucleotide sequence, through the guide RNA, in CRISPR-Cas. Thus, I'd "expect" all targeted sites to be this guide RNA sequence. This should also apply to off-target sites. Therefore, I'd assume p_g to be sequence position specific, namely 1.0 for the nucleotide of the guide and 0 otherwise - maybe add some uncertainty to avoid 0 probabilities, i.e. 0.97, 0.01, 0.01 and 0.01 for the four bases.

c) I have even greater concerns with the authors' similarity metric to compare multiple off-target site "patterns". It is purely based on relative entropy, which is independent of sequence content. This could lead to extremely different sequence patterns that are considered identical. For example, take the pattern of Figure 2, panel (a). Entropy of positions 12 to 20 is basically at maximum, i.e. only these bases have been observed. If we replace the bases CCAGAAGCT with TTTTTTTTA, which is obviously very different, the entropy wouldn't change at all!

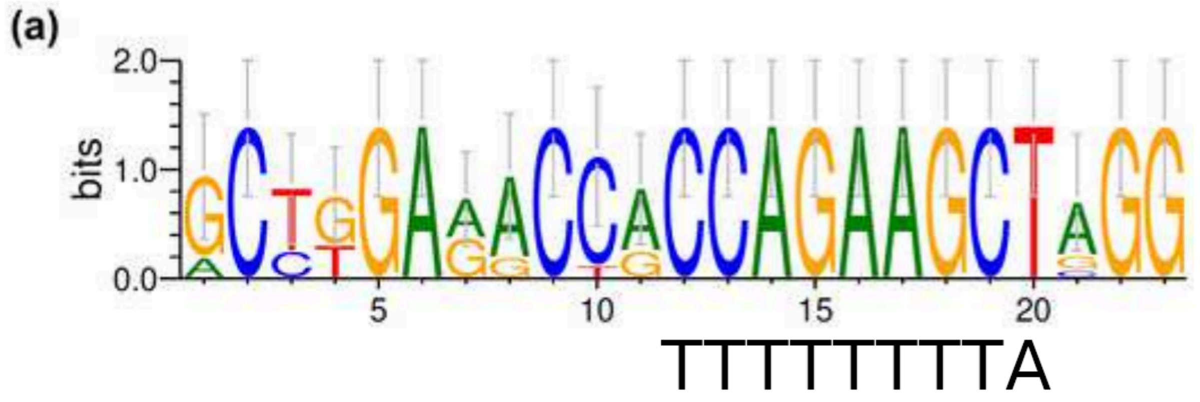

I am therefore convinced that all downstream conclusions are flawed!

Minor issues:

d) The presentation of the number of studies / datasets is inconsistent and hard to understand. Authors speak about one "plus five additional human datasets" (line 132) in the Materials and Methods section, "three independent studies" (line 349) in the Discussion but present 234 "datasets" and 2827 "digested sequences" in Table 1.

e) The pattern construction and comparison strongly reminds me of transcription factor binding site prediction and the rich research body on this topic. Authors might read up methodology in this multiple decades ago established field to carry over best practices to their application.

f) The table in Figure 1 and the stacked bar-chart is duplicating information. Supplementary Table 3, on the other hand, holds valuable information which I would not hide in the supplement but directly present in Figure 1. Maybe even merge with supplementary Figure 1 or create a multi panel figure.

g) I think statements like "These results align with our visual observations, confirming that the sequence patterns for FANCF-site6 were similar between the two enzymes, whereas those for HEKsite4 were dissimilar." (line 279) are probably invalidated with a proper similarity metric and I don't like the fact that authors only evaluated their metric "visually" on just two examples. A more thorough investigation should have brought up the sequence independence problem - see my issue c).

h) I do not directly see how one could test the significance of the similarity, e.g. line 291, without a proper background model of pattern similarities, a density function or a permutation approach. Depending on the clustering of off-site target sequences and their multiple sequence alignment, it is likely that we have a high false negative rate and thus a very incomplete ranked list of patterns.

In conclusion, my recommendation is to only accept this manuscript if authors provide **major revisions**. The methodology is flawed, but the general research question is worth being pursued.
