# Supplementary material for: Off-target sequence variations driven by the intrinsic properties of the Cas–sgRNA–DNA complex in genome editing
Source: PLoS One. 2025 Jul 18;20(7):e0328905. doi: 10.1371/journal.pone.0328905 (PMC12273960; doi:10.1371/journal.pone.0328905)
Supplement: Attachment — Submitted filename: Response_to_reviewers_auresp_2.docx [file pone.0328905.s006.docx]

**Response to Reviewers**

We are truly grateful to the reviewers for re-reviewing our revised manuscript. We have made the necessary revisions and provide a point-by-point response below.

>Reviewer #1: In this revised version of the manuscript, the authors addressed the comments of the reviewers, however some points require further considerations.

>

>Major points:

>23 "We developed a method to assess sequence pattern similarity and diversity between off-target sites. This method is based on a comparison of ordered relative entropy values for aligned target sequences, and it was compared with two other methods on the basis of Euclidean distance and the Pearson correlation coefficient. The three methods demonstrated clear correlations, indicating their validity."

>366 "These results indicate that the three methods could consistently detect similar or divergent sequence patterns for the 238 pairs"

>It is unclear what is the advantage of the new method based on ordered relative entropy.

The proposed method provides statistical significance in measuring the similarity between sequence patterns, which can help researchers more easily identify biologically relevant patterns. This point has been addressed in the abstract and in the discussion.

>130 "In this study, we first assessed the performance of CRISPOR's computational predictions by comparing them with off-target sites identified through the experiment-based GUIDE-seq method. Additionally, since a number of other novel methods have also been developed, CRISPOR’s predictions were further validated using CRISOT, which outperforms other in silico methods [25]"

>192 "Off-target sequences identified by GUIDE-seq and predicted by CRISPOR were utilized for computing CRISOT scores [25]"

>If CRISOT outperforms other in silico methods, including CRISPOR, the prediction analyses should have been done using CRISOT; and also the 'Comparison between GUIDE-seq data and computational predictions" of 3.2 and Fig.1. Instead, CRISOT was used only to score the off-targets in common between CRISPOR and GUIDE-Seq. It is also possible that CRISOT could have found off targets undetected by both the other two methods.

In this section, our primary aim was to highlight general issues in off-target prediction. CRISPOR is widely used in the field; to our knowledge, publications related to the CRISPOR methodology have been cited over ten times more frequently than those for other methods. Therefore, we focused primarily on this method. Furthermore, the predictive performance of CRISOT, regarding both true positives (active sites) and false positives (inactive sites), was already evaluated by its original authors (Fig. 4, Chen et al., 2023), and we would like to avoid redundant data analysis. Nevertheless, we understand the reviewer's concern and have now included additional data on genome-wide prediction (S2 Fig.), which help readers understand the general performance of the CRISOT method.

>357 "The findings presented in this study provide a foundation for developing a framework aimed at enhancing computational methods for off-target sequence prediction. While we can perform large-scale data analysis to discover the characteristics of off-target sequences, more experimental data have been produced using GUIDE-seq and other effective methods. It is expected that further analysis of such data will lead to the identification of precise biological mechanisms of off target mutation generation, and if these mechanisms are validated experimentally, this information can be used to improve computational prediction models in the future".

>The contribution of the present work is not clearly stated.

To emphasize the significance of our study, we have revised the relevant section accordingly.

>Fig.4: Because a) is determined by only 2 sequences, and b) by 144 sequences, could the differences between a) and b) potentially disappear obtaining and analysing the same number of sequences for both the enzymes?

This is an important point. The number of detected off-target sequences varied significantly in some cases, as previously reported (Fu et al., 2013; Zhou et al., 2022). However, such differences can themselves be considered reflective of underlying sequence pattern divergence. This issue is now addressed and discussed in 4.1.

>Minor points:

>

>283 "Table 2. Recall, precision and F1 score for different mismatch numbers." The authors should specify the method.

We have revised the sentence as follows:

Table 2. Recall, precision and F1 score for different mismatch numbers. True positives, false negatives, and false positives were defined on the basis of the results from GUIDE-seq and CRISPOR.

>355 "To examine the efficacy of the aforementioned method, large-scale comparisons with two additional methods based on the Euclidean distance (ED) and Spearman's correlation coefficient (PCC) were performed".

>I think the authors mean Pearson correlation coefficient, instead of Spearman's.

Thank you for pointing this out. The typo has been corrected.

>357 "The findings presented in this study provide a foundation for developing a framework aimed at enhancing computational methods for off-target sequence prediction. While we can perform large-scale data analysis to discover the characteristics of off-target sequences, more experimental data have been produced using GUIDE-seq and other effective methods. It is expected that further analysis of such data will lead to the identification of precise biological mechanisms of off target mutation generation, and if these mechanisms are validated experimentally, this information can be used to improve computational prediction models in the future".

>The part: "While we can perform.... more experimental data have been produced" is unclear.

The indicated sentence has been revised for clarity.

>Reviewer #2: I am limiting myself to the three major issues I have raised before:

>

>@a: Regarding the suggested extension of acquiring additional raw data, authors state that "it is not feasible for us to repeat this process".

As mentioned in our previous response, in addition to the practical challenges of restarting the entire analyses from data collection stage, the amount of the data used in this study is sufficiently large and diverse to support our conclusion. While using the most up-to-date datasets is ideal, we hope it is understandable that substantial data analysis and related tasks require time, and as a result, the dataset used may not always reflect the latest available data.

>Regarding software tools: authors explain in their response that "primary aim was to illustrate the current state of available standard programs". They did add CRISTOR in their manuscript, but I miss back to back evaluation results. Furthermore, I still strongly disagree that it suits the claimed aim if authors just present / discuss / analyse two of - in their words - "numerous in silico prediction tools". I would have at least expected a comparison - maybe a table - of strength and weaknesses of these "numerous" tools.

>In conclusion, my issue is not appropriately addressed.

As indicated in the title, our primary goal was to elucidate the general characteristics of off-target mutations. We also believe that a deeper understanding of these features will contribute to improving prediction tools. While we appreciate the reviewer's suggestion, presenting a comprehensive evaluation of all currently available tools would, in our view, shift the focus of the paper and potentially mislead readers regarding its core objective.

>@b: I fully agree on the author's assumption: "if two observed patterns deviate in different directions from the random baseline, such differences can be detected using an appropriate statistical method", however, I think they model the starting point of potential deviations incorrectly. It is way more likely to deviate in different directions if you are in the center of a chessboard compared to starting at the top left corner. Random sequences would be the center, the actual sequence pattern is - in my view - much more at the borders.

>To be clear, I am not criticizing the metric here, but the assumption of the background model. The fact that other metrics correlate, does not counter my argument, as they would "suffer" from the very same background issue.

>I feel we have a misunderstanding here and thus the core of my concern was not addressed.

Our focus here is on differences between sequence patterns, and the probability of distinct patterns producing highly similar relative entropy orders is negligibly small. While we acknowledge the concern, it is difficult for us to conceptualize how three independent metrics could all be affected by the same background issue, as the nature of the issue itself remains unclear.

We believe the observed consistency among the three metrics provides a strong indication of the reliability of our results. Nevertheless, to support further investigation and transparency, we have made all WebLogo data publicly available at https://github.com/taitoh1970/ge_off_targets, as noted in 2.4.

>@c: Looking at the scatter plots of S3 and S4, I feel the results are not very convincing. The computed correlation is high for all data, however, individual data points show huge dissimilarities. The fact that these deviations are not even more pronounced is properly due to the underlying data, i.e. I agree that "the extreme cases mentioned by the reviewer may be uncommon". From the reply to my concern, I cannot clearly see if the authors agree with my assessment, that their metric is sequence independent or not.

>Leaving out the theoretical considerations, author's have demonstrated that their metric is roughly on par with state of the art alternatives, thus, my open question might be relevant to the reader.

>

>In conclusion, I am not fully satisfied with how the authors addressed my concerns and would like to see further editions on the manuscript.

First, we acknowledge that the proposed method assesses sequence patterns indirectly through relative entropy values, and we have now clarified this point in 4.1. Second, we recognize the reviewer's concern regarding the scatter plots, when Euclidean distances are large and correlation coefficients are small. Therefore, we introduced a conservative categorization of data points as "semi-divergent" when either the SRCC or the ED alone was insufficient to indicate strong similarity. This approach ensures that only patterns with consistent evidence across multiple metrics are classified as "similar," which we believe strengthens the reliability of the analysis. On the basis of this conservative criterion, we consider the results to be reasonably robust.

Reviewer #3: The authors have addressed all my comments, and thus I have no more comments on the revised article.

We sincerely appreciate the reviewer's thoughtful evaluation and are grateful for the acceptance of the revised manuscript.
